# Determining Factors in the Implementation of Biosecurity Measures by Hospital Nurses in Piura, Peru

**DOI:** 10.3390/nursrep14030158

**Published:** 2024-08-26

**Authors:** Luz Mirella Agurto Córdova, Danicsa Karina Espino Carrasco, Briseidy Massiel Santa Cruz Espino, Mayury Espino Carrasco, Cindy Vargas Cabrera, Royer Vásquez Cachay, Lady Dávila Valdera, Edson David Valdera Benavides, Roque Valderrama Soto

**Affiliations:** 1 Campus Piura, Facultad Ciencias de la Salud, Escuela de Enfermería, Universidad César Vallejo, Piura 20001, Peru; lagurtoco@ucvvirtual.edu.pe (L.M.A.C.); ldavilaval@ucvvirtual.edu.pe (L.D.V.); vsotoro@ucvvirtual.edu.pe (R.V.S.); 2 Campus Pimentel, Facultad de Ciencias de la Salud, Escuela de Medicina Humana, Universidad Señor de Sipán, Chiclayo 14000, Peru; sespinobriseidm@uss.edu.pe (B.M.S.C.E.); ecarrascom@uss.edu.pe (M.E.C.); vcabrera@uss.edu.pe (C.V.C.); vcachayr@uss.edu.pe (R.V.C.); 3 Campus Lambayeque, Administrativas y Contables, Facultad de Ciencias Económicas, Escuela de Administración, Universidad Nacional Pedro Ruiz Gallo, Lambayeque 14013, Peru; evalderab@unprg.edu.pe

**Keywords:** biosafety, nosocomial infections, nursing staff, extrinsic factors, intrinsic factors

## Abstract

Nosocomial infections are a significant cause of morbidity, mortality, and increased treatment costs in hospitals. This study aimed to analyze the factors determining the implementation of biosafety measures by the nursing staff of four hospitals in Piura via a structural equation modeling (SEM) approach. A total of 215 nurses from various hospitals in the region participated by completing an online survey. The results demonstrated that extrinsic factors (FEX) positively influence the implementation of biosafety measures (BIOM) (β = 0.319 ***), as do intrinsic factors (FINT) (β = 0.520 **). Furthermore, intrinsic factors mediate the relationship between extrinsic factors and the implementation of biosafety measures (β = 0.443 ***). In conclusion, this study provides a deeper understanding of biosafety dynamics in healthcare settings and lays the groundwork for the development of customized interventions and ongoing training programs that ensure the optimal implementation of biosafety measures in hospitals.

## 1. Introduction

Nosocomial infections are a leading cause of morbidity, mortality, and increased treatment costs in hospital settings and commonly occur in the bloodstream, lower respiratory tract, and surgical sites [1,2]. Hospital management and architectural design play a significant role in controlling these infections, with factors such as appropriate equipment placement, air ventilation, and room layouts influencing infection rates [3]. In this context, biosafety measures in hospitals are crucial for preventing nosocomial infections, as demonstrated by several studies. Knowledge of biosafety among nursing staff is moderate, and important gaps have been detected in administrative controls, standard microbiological practices, and facility design [4]. Additionally, factors influencing the adoption of biosecurity measures by nursing professionals include personal and institutional factors, with unfavorable factors such as the lack of specialized studies and training in biosecurity [5]. Strengthening awareness of biosecurity among medical microbiology researchers is essential to ensure the safety of both the researcher and the laboratory environment [6], whereas adherence to precaution and prevention measures, collectively interpreted as biological safety (biosecurity), is crucial for the staff working with pathogenic and infectious agents in clinical laboratories [7].

Furthermore, statistical data highlight the importance of addressing this public health issue. Globally, the prevalence of nosocomial infections varies by region. In Germany, a national prevalence study revealed that approximately 5.3% of patients acquired a nosocomial infection [8], while in the United States, these infections are the sixth leading cause of death [9]. The most common types of nosocomial infections include catheter-associated bacteremia, ventilator-associated pneumonia, surgical site infections, and catheter-associated urinary tract infections [10]. However, the prevalence of specific infections varies by region. For example, in France, the most common infections are urinary tract infections, pneumonia, surgical site infections, and bloodstream infections [8], whereas in Mazandaran Province, Iran, a higher incidence of infections due to pneumonia, urinary infection, surgical incisions, and burns has been identified [11].

A study conducted in a Chinese hospital over a ten-year period reported an infection rate of 2.18%, with respiratory system infections being the most common, followed by bloodstream and genitourinary system infections [12]. Additionally, the prevalence of antimicrobial use in acute care hospitals in Canada increased between 2002 and 2009 and then stabilized between 2009 and 2017, indicating possible changes in infection control practices over time [13].

Various risk factors contribute to the high incidence of nosocomial infections, such as a previous intensive care unit (ICU) stay, surgery, invasive device placement, and prior use of specific antibiotics [12]. Furthermore, the pathogens most frequently isolated from these infections include *Staphylococcus aureus*, *Acinetobacter baumannii*, *Klebsiella pneumoniae*, *Pseudomonas aeruginosa*, and *Escherichia coli* [14].

Nursing staff play a crucial role in implementing biosecurity measures, significantly impacting patient care quality. Various studies have shown that nursing professionals are aware of the importance of these measures, but their adherence to them is limited [15,16,17]. Factors such as youth, a lack of specialized studies, and insufficient training in biosecurity contribute to the unfavorable application of biological safety measures by these personnel [5,18].

However, nursing interventions can positively impact the level of knowledge about biosecurity norms, especially in the care of patients with infectious diseases such as COVID-19 [4,18]. However, problems related to the availability of personal protective equipment (PPE), such as alcohol-based hand sanitizers, and gaps in knowledge about the properties of alcohol and occupational hazards have been identified as obstacles to full compliance with biosafety guidelines [17].

Additionally, nursing students recognize biosecurity measures as a set of norms for the protection of workers and patients, particularly during the pandemic, but lack the confidence to address them effectively [19].

In this context, the present study aimed to analyze the determining factors in the implementation of biosecurity measures by nursing staff at four hospitals in Piura. Identifying these factors will allow the development of effective strategies to improve adherence to biosecurity measures and, consequently, optimize the quality of care provided to patients. This study is justified by the pressing need to strengthen biosecurity practices in hospital settings, especially in regions where nosocomial infections pose a significant challenge to public health. Through a multidisciplinary approach that includes evaluating both personal and institutional aspects affecting the implementation of these measures, we hope to provide a comprehensive overview that contributes to decision-making and the implementation of more effective health policies.

The results of this study are expected to highlight the importance of ongoing and specialized training in biosecurity for nursing staff, as well as the need to improve the infrastructure and resources available for implementing these measures. Furthermore, this study aims to underscore the importance of creating a safe culture that promotes individual and collective responsibility in preventing nosocomial infections.

Therefore, this study contributes significantly to the literature by providing specific evidence on the factors influencing the implementation of biosecurity measures in a specific hospital context. Additionally, the findings could inform the development of targeted training programs and improvements in institutional policies, with the potential to be applied in other similar settings to increase patient and health staff safety.

## 2. Literature Review

### 2.1. The Main Biosafety Measures in the Hospital Environment

Hand hygiene is one of the most critical measures for preventing the cross-transmission of microorganisms and plays a crucial role in the prevention of nosocomial infections [20]. Although most healthcare professionals are knowledgeable about hand hygiene, one study reported that proper handwashing practices were not adequately followed, highlighting the need for effective training and improved practices [20].

On the other hand, the availability of biosafety facilities and equipment, such as gloves, goggles, masks, and lab coats, is essential to keep healthcare personnel and the surrounding environment safe from infectious diseases [4]. These protective measures are essential to ensure laboratory safety and prevent the spread of infection.

Moreover, hospital waste, including potentially hazardous and infectious materials, poses a significant risk to human health and the environment [21,22]. Proper management of these wastes is crucial for preventing nosocomial diseases and environmental damage [21,22]. Biosecurity programs are necessary to assess the risks associated with medical waste accurately and to implement appropriate management strategies [23].

The correct disposal of hospital waste is of paramount importance because of its infectious and hazardous characteristics [24]. Effective segregation protocols significantly reduce the generation of biomedical waste, underscoring the importance of segregation at waste production sources [25]. Furthermore, it is essential to pay more attention to the onsite collection and safe storage of genotoxic wastes in hospitals [26].

### 2.2. Factors Influencing the Implementation of Biosafety Measures by Nurses

The implementation of biosecurity measures by nursing staff is influenced by a combination of intrinsic and extrinsic factors. Intrinsic factors pertain to the individual characteristics of the nursing staff, such as their knowledge, attitudes, and perceptions, whereas extrinsic factors relate to the environment and conditions in which they work.

Among the intrinsic factors, knowledge and training play crucial roles in the implementation of biosecurity measures. A study revealed that 57.14% of nurses acquired knowledge about biosecurity through training courses [27]. Additionally, the attitudes and perceptions of nursing staff influence their behavior. Nursing students who demonstrated a positive level of knowledge about biosecurity measures, waste classification, and hand hygiene were more likely to implement these practices [28]. Nurses’ attitudes toward computer security are also positively related to compliance with information security policies and competence in information handling [29].

For extrinsic factors, training and the availability of equipment are essential for the effective implementation of biosecurity measures. A survey revealed that 79.0% of nursing professionals had not received adequate training or considered it insufficient, and 69.3% reported a lack of personal protective equipment during work [30]. Continuing education has been identified as a factor that contributes to increasing nurses’ awareness of the necessary safety in the profession, which could reduce the cost-effectiveness of hospitalizations [31]. Moreover, organizational support, including backing from the administration and guidance for action, significantly influences safety behavior in healthcare facilities [32].

Therefore, both intrinsic and extrinsic factors play significant roles in the implementation of biosecurity measures by nursing staff. It is crucial to address these factors comprehensively and provide adequate training, personal protective equipment, continuous education, and organizational support to ensure the effective application of these critical safety measures in the hospital setting.

### 2.3. Scientific Support of the Research Hypotheses

**Figure 1:**
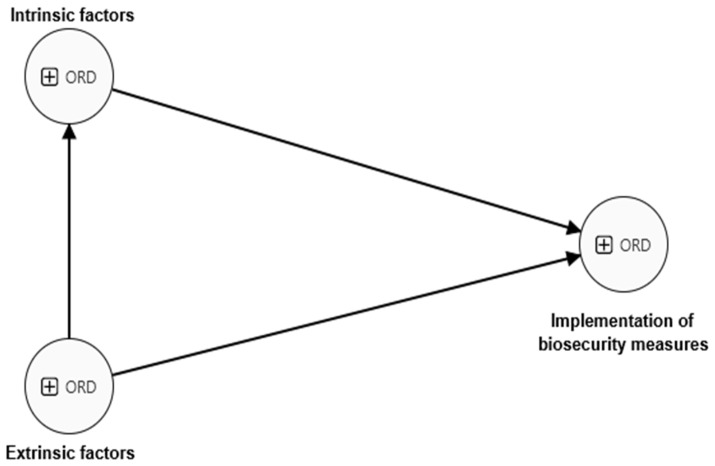
Proposed research model. Note: The circles of the proposed model appear + ORD and represent that it is composed of items with an ordinal scale.

Figure 1 shows the proposed research model. Multiple studies support the hypothesis that extrinsic factors, those external to the individual, significantly influence the implementation of biosecurity measures by medical personnel. A study conducted in Peru among nursing professionals revealed that both personal and institutional factors influence the adoption of biological safety measures [5]. Unfavorable institutional factors include the lack of an epidemiology office and the absence of continuous supervision in the application of biosecurity. This suggests that institutional policies and practices play a crucial role in promoting or inhibiting adherence to biosecurity measures. Furthermore, the importance of biosecurity training for medical staff and students has been highlighted in various studies [4,33,34]. The absence of adequate training programs and gaps in knowledge about biosecurity measures can hinder their effective application. This implies that the availability and quality of education and training, which are extrinsic factors, have a direct effect on the biosecurity practices of medical staff. Another extrinsic factor that influences the adoption of biosecurity measures is the framework of policies and government interventions. A study conducted in Belgium evaluated the impact of policy interventions on the adoption of biosimilars and revealed a variable and limited impact [35]. This suggests that a holistic policy approach is needed to foster adherence to biosecurity practices. Additionally, the characteristics of providers and patient demographics can influence the adoption of biosecurity measures. A study in the U.S. revealed that office-based providers and those with larger practices and a greater proportion of certain patient demographics were more likely to adopt biosimilars [36]. This finding indicates that factors related to the practice environment and patient population, which are extrinsic to the individual, can shape decisions on the implementation of biosecurity measures. Consequently, extrinsic factors may influence the application of biosecurity measures by nursing staff. Thus, the following hypothesis is formulated:

**Hypothesis** **1.** *Extrinsic factors (FEXs) influence the application of biosecurity measures (BIOMs) by nursing staff*.

Intrinsic factors, which are inherent to the individual, play a crucial role in the implementation of biosecurity measures by medical staff. Evidence from various studies supports this hypothesis. Initially, a study on nursing professionals identified several personal factors that influence the adoption of biological safety measures [5]. It was found that characteristics such as being a young adult, a lack of specialized studies, and insufficient training in biosecurity had a negative impact on the application of these measures. This suggests that individual attributes and the level of education and training, which are intrinsic factors, play a significant role in adherence to biosecurity practices. Moreover, knowledge about biosecurity was identified as a key intrinsic factor influencing the application of biological safety measures. A study revealed that the level of knowledge about biosecurity among laboratory staff was generally intermediate, and only a small percentage had participated in formal training courses [33]. The level of knowledge increased with work experience, highlighting the importance of continuous training and exposure to improve adherence to biosecurity practices. The importance of education and training in biosecurity was also highlighted in a study on academic medical centers [34]. A solid foundation in the principles of biosecurity was found to be crucial for both clinical and research work, and the need for training to increase compliance with safety measures was emphasized. This implies that the knowledge and skills acquired through education and training, which are intrinsic factors, have a direct impact on the application of biosecurity practices. Furthermore, a survey of clinical and public health laboratories identified deficiencies in biosecurity practices, including the lack of specific patient information for risk assessment and incomplete biosecurity elements in some laboratories [37]. These deficiencies can be attributed to intrinsic factors such as a lack of knowledge, skills, and awareness of proper biosecurity procedures. Consequently, intrinsic factors may influence the implementation of biosecurity measures by nursing staff. Thus, the following hypothesis is formulated:

**Hypothesis** **2.** *Intrinsic factors (FINT) influence the implementation of biosecurity measures (BIOM) by nursing staff*.

Multiple studies suggest that both extrinsic and intrinsic factors influence the application of biosecurity measures by nursing staff and that the influence of extrinsic factors may be mediated by intrinsic factors. With respect to extrinsic factors, the work environment has a significant effect on the adoption of biosecurity practices. Office-based providers adopted biosecurity measures earlier and more quickly [36], suggesting that workplace characteristics, such as resource availability and institutional support, influence the implementation of these measures. Moreover, facility design and administrative controls compromise biosecurity performance in some laboratories [4], further highlighting the importance of environmental factors. However, the influence of these extrinsic factors appears to be mediated by intrinsic factors, such as the personal and professional characteristics of nursing staff. A study revealed that nursing professionals with unfavorable personal factors, such as being young adults, lacking specialized studies, and not having training in biosecurity, were less likely to implement biological safety measures effectively [5]. This suggests that individual attributes and the level of education and training, which are intrinsic factors, play a role in translating external influences into actual biosecurity behaviors. Furthermore, a study using the biosecurity incident response competence scale for nurses revealed significant differences in scores on the basis of intrinsic characteristics such as years of work experience, degrees, and participation in biosecurity training [38]. This finding implies that intrinsic factors shape the competence and ability of nursing staff to respond to external demands related to biosecurity. The mediating influence of intrinsic factors was also evidenced in a study conducted in Morocco, which reported that the level of knowledge about biosecurity among laboratory staff increased with work experience [33]. This suggests that professional development programs and training, which are extrinsic factors, impact biosecurity practices by enhancing individuals’ knowledge and skills. Consequently, it can be inferred that extrinsic factors influence the implementation of biosecurity measures, and this influence is mediated by intrinsic factors by nursing staff. Thus, the following hypothesis is formulated:

**Hypothesis** **3.** *Extrinsic factors influence the implementation of biosecurity measures, and this influence is mediated by intrinsic factors by nursing staff*.

## 3. Materials and Methods

The research followed a quantitative approach of exploratory and explanatory types because the purpose was to analyze the determining factors in the implementation of biosecurity measures by the nursing personnel of four hospitals in Piura. In addition, the study has a nonexperimental design and is cross-sectional since it does not aim to manipulate the variables under study, and the measurements were carried out over a single period of time.

### 3.1. Participants

A total of 215 nurses working in various hospitals in the Piura region of Peru participated. A nonprobabilistic convenience sampling method was employed, where staff participated freely and voluntarily after providing informed consent. According to Table 1, of the total nursing staff surveyed, 23.8% (51 participants) were men, and 76.16% (163 participants) were women. The majority of respondents were between 25 and 35 years old, representing 76.5% (164 participants) of the sample. A smaller proportion of respondents, 10.3% (22 participants), were between 36 and 40 years old. The age brackets of 41–45 years, 46–50 years, and 51–55 years each represented 4.3% (9 participants for each age bracket) of the population, and only 0.4% (1 participant) were in the age bracket of 56–60 years.

With respect to service time, 26.05% (56 participants) had been working for up to 1 year, and 15.81% (34 participants) had been working for up to 2 years. A significant portion, 27.44% (59 participants), had a service time of up to 3 years. Fewer nurses, 14.42% (31 participants), had been working for up to 4 years, and 16.28% (35 participants) had a service duration of more than 5 years.

### 3.2. Data Collection Instruments

To structure the data collection instrument, a comprehensive review of the literature was conducted to identify factors such as extrinsic factors (FEX), intrinsic factors (FINT), and the implementation of biosafety measures (BIOM).

An online form consisting of two sections was subsequently structured. The first section included sociodemographic questions such as questions about gender, age, and service time. In the second section, 29 items were organized: 4 items pertained to the FEX, 5 items to the FINT, and 20 items to the BIOM. The response scale for the items in the second section was a 5-point Likert-type scale ranging from (1) strongly disagree to (5) strongly agree.

### 3.3. Procedure and Data Analysis

The data were collected through the application of an online survey in the months of August and October 2023. The average time of application of the form was 15 min. A total of 215 responses were collected from participants who agreed to participate voluntarily by completing the informed consent form.

Structural equation modeling (SEM) was used to analyze the data with Smart-PLS statistical software, which employs the partial least squares (PLS) technique to test the theoretical model. Reliability was evaluated via the analysis of factor loadings, Cronbach’s alpha coefficient, and composite reliability (CR), whose values were above 0.7 (Table 2 and Table 3). The average variance extracted (AVE), whose values were greater than 0.5, was used to evaluate convergent validity (Table 3). Similarly, to evaluate discriminant validity, the criterion of [39] was followed, where the root of the AVE of each construct was analyzed so that its values were not greater than the correlations of all the other constructs and the specific construct.

## 4. Results

### 4.1. Results of the Measurement Model

Table 2 shows the results of the factor loadings. According to [40], it is recommended that the factor loadings exceed 0.5, which in the study shows that all the items exceed this threshold. On the other hand, the values of the variance inflation factor (VIF) for all the items range between 2 and 3.5, indicating moderate and acceptable correlations.

Table 2 presents the results of the reliability and discriminant and convergent validity tests. With respect to Cronbach’s alpha (α) and composite reliability (CR) for measuring the reliability of the latent variable, following the criterion of [41], values above 0.70 are considered adequate; as illustrated in Table 2, all the constructs exceed this threshold. The average variance extracted (AVE) is used to determine convergent validity, and according to [42], values above 0.50 are acceptable; similarly, all the constructs in the model exceed this threshold. The values of the determination coefficient (R^2^) indicate that FEX and FINT explain 65.6% of the variation in BIOM, whereas FEX explains 72.5% of the variation in FINT.

Finally, discriminant validity was calculated following the criterion of [39], where for discriminant validity to exist, the square root of the AVE (numbers on the diagonal) must be greater than the correlations with other constructs (numbers outside the diagonal in the same row and column), and as observed in the study, all the constructs met this criterion.

### 4.2. Contrasting the Research Hypotheses

**Figure 2:**
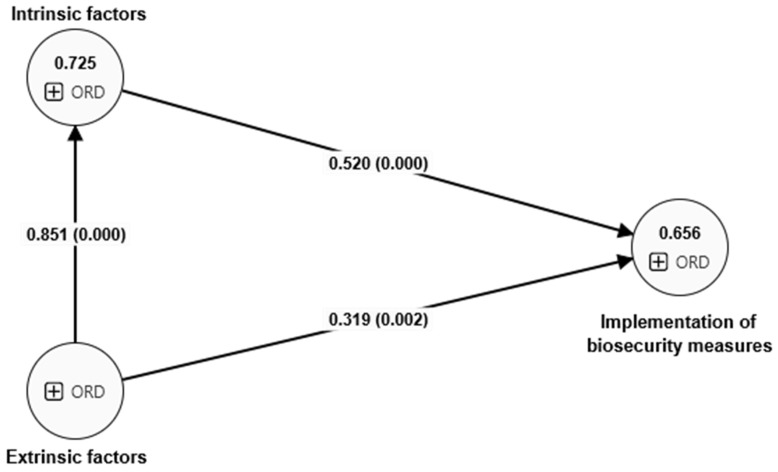
Resolved research model Note: At the intersections of the relationship lines are the path coefficients on the left and the *p* values on the right (inside the parentheses).

Table 4 and Figure 2 show the standardized path coefficients (B), *p* values, confidence intervals, and standard deviations (SDs). H_1_ has a path coefficient of B = 0.319 and a *p* value of 0.000 < 0.001, so it is accepted. H_2_ has a path coefficient (B = 0.520) and a *p* value of 0.002 < 0.05, so it is accepted. Finally, with respect to the mediation hypothesis (H_3_), it presents a path coefficient (B = 0.443) and a *p* value of 0.000 < 0.001, so it is accepted.

## 5. Discussion

This study aimed to analyze the factors determining the implementation of biosecurity measures by nursing staff at four hospitals in Piura. To test the research hypotheses, a structural equation model (SEM) was formulated whose constructs met the criteria for reliability and convergent and discriminant validity.

With respect to hypothesis 1, the results show that the extrinsic factors (FEX) significantly influence the implementation of biosecurity measures (BIOM), with a path coefficient of 0.319 and a *p* value of 0.000, indicating a positive and significant relationship. This result confirms the importance of the work environment, including the availability of resources and institutional support, in promoting safe practices among nursing staff. This finding is consistent with the literature, suggesting that institutional policies and practices play a crucial role in promoting adherence to biosecurity measures [5,35]. The adequacy of facilities and the availability of personal protective equipment are essential for the effective implementation of biosecurity measures, underscoring the need for solid organizational support.

Hypothesis 2, which posits a positive influence of intrinsic factors (FINT) on the implementation of biosecurity measures, was supported by a path coefficient of 0.520 and a *p* value of 0.002. This result highlights the relevance of individual characteristics, such as the knowledge, attitudes, and perceptions of nursing staff, to their behavior toward biosecurity. The findings emphasize the importance of training and continuous education in improving adherence to biosecurity practices, in line with previous studies that emphasize the role of training in biosecurity [33,34]. This result underscores the need for targeted training policies that improve the knowledge and skills of nursing staff in biosecurity.

Finally, the mediation hypothesis (H_3_) was also confirmed, indicating that intrinsic factors mediate the relationship between extrinsic factors and the implementation of biosecurity measures, with a path coefficient of 0.443 and a *p* value of 0.000. This implies that although the work environment and institutional support are crucial, their effectiveness in promoting biosecurity is conditioned by the individual characteristics of the nursing staff, such as their training, knowledge, and attitudes toward biosecurity. This result underscores the importance of an integrated approach that combines improvements in the work environment with strategies aimed at the professional development and training of nursing staff.

The findings of this study underline the complexity of implementing biosecurity measures in hospital settings, highlighting the influence of both extrinsic and intrinsic factors [43]. These results reinforce the need for multifaceted strategies that address both the work environment and the professional development of nursing staff. Continuous training, institutional support, and infrastructure improvements are key to the effective implementation of biosecurity measures, which, in turn, will help improve the quality of patient care and reduce the risk of nosocomial infections. The implementation of these strategies requires organizational commitment and well-coordinated public health policies to ensure a safe working environment for both healthcare staff and patients.

## 6. Conclusions

This study, conducted in a hospital in Piura on the determining factors in the implementation of biosafety measures by nursing staff, shed light on crucial aspects impacting the safety of patients and healthcare workers. Through a rigorous quantitative analysis, significant intrinsic and extrinsic factors influencing the adoption of these critical practices were identified.

The findings not only support previous theories but also provide new insights for a deeper understanding of biosafety dynamics in healthcare settings. The relevance of contextual elements, such as the work environment and institutional support, as well as individual aspects, such as staff knowledge level and attitudes, were confirmed [44]. The evidence of a dynamic interaction between these factors highlights the need for comprehensive approaches that address structural improvements and promote ongoing professional development.

A novel contribution of this study is the identification of the mediating role of intrinsic factors in the relationship between extrinsic factors and the implementation of biosafety measures. This suggests that the interventions aimed at optimizing these practices should be customized to meet the specific training and motivation needs of nursing staff.

Furthermore, the crucial role of continuous training in improving adherence to biosafety protocols was reaffirmed, with challenging strategies focused solely on infrastructure improvements or standardized guidelines. This outcome advocates for tailored professional development and education programs designed for hospital contexts, adapting to the changing requirements of staff and innovations in biosafety.

Finally, this study provides the scientific community with a validated and reliable measurement tool. This instrument represents a valuable resource for future studies, allowing replications and comparisons in diverse healthcare contexts. This methodological contribution has the potential to drive standardized biosafety research, facilitating the development of evidence-based strategies to improve practices in healthcare settings.

In summary, this study not only validates existing theories but also opens new horizons for future research. This emphasizes the importance of considering the dynamic interaction between contextual and individual factors, as well as the need for personalized interventions and continuous training programs [45]. These findings lay the groundwork for developing more effective strategies that ensure the optimal implementation of biosafety measures in hospital environments, thereby contributing to the safety and well-being of both patients and health professionals.

## 7. Limitations and Future Studies

Although this study provides valuable insights, it has limitations, including its focus on a single hospital and its reliance on self-reported data, which may introduce response biases. Future research could expand to multiple hospital settings to validate these findings in a variety of clinical contexts. Moreover, the study population only belonged to four hospital centers, and the sample was selected through nonprobabilistic sampling, making it likely that the study results may not be generalizable to similar populations in other contexts.

Therefore, investigating the impact of specific interventions designed to improve the identified extrinsic and intrinsic factors is suggested, thereby providing a more precise guide for biosafety policies.

## Figures and Tables

**Table 1:** Sociodemographic characteristics of the study population (*n* = 215).

	Fi	%
Gender		
Male	51	23.8
Female	163	76.16
Age		
25–30	78	36.5
31–35	86	40. 2
36–40	22	10. 3
41–45	9	4.3
46–50	9	4.3
51–55	9	4.3
56–60	1	0.4
Service time		
Up to 1 year	56	26.05
Up to 2 years	34	15.81
Up to 3 years	59	27.44
Up to 4 years	31	14.42
More than 5 years	35	16.28

Note: Own elaboration.

**Table 2:** Factor loadings and collinearity statistics.

Items		Construct	Outer Loadings	VIF
I consistently perform hand hygiene before and after patient contact.	BIOM1 <- BIOM	Implementation of biosecurity measures (BIOM)	0.790	2.716
I always wear appropriate personal protective equipment when needed.	BIOM10 <- BIOM		0.766	2.047
I properly dispose of medical waste according to hospital guidelines.	BIOM11 <- BIOM		0.790	3.146
I follow correct protocols for handling and disposing of sharp objects.	BIOM12 <- BIOM		0.769	2.203
I maintain a sterile field during invasive procedures.	BIOM13 <- BIOM		0.742	2.678
I regularly clean and disinfect frequently touched surfaces in my work area.	BIOM14 <- BIOM		0.770	2.613
I follow isolation precautions for patients with infectious diseases.	BIOM15 <- BIOM		0.789	2.091
I properly handle and store potentially infectious specimens.	BIOM16 <- BIOM		0.808	3.996
I educate patients and visitors about necessary biosecurity measures.	BIOM17 <- BIOM		0.810	3.164
I report any breaches in biosecurity protocols to the appropriate authority.	BIOM18 <- BIOM		0.816	2.292
I participate in regular biosecurity training and updates.	BIOM19 <- BIOM		0.764	2.603
I properly use and maintain biosecurity equipment (e.g., biological safety cabinets).	BIOM2 <- BIOM		0.733	2.104
I follow correct procedures for decontamination of equipment and work areas.	BIOM20 <- BIOM		0.856	2.859
I adhere to protocols for safe handling and transport of biological materials.	BIOM3 <- BIOM		0.744	2.031
I implement proper respiratory hygiene and cough etiquette.	BIOM4 <- BIOM		0.715	3.475
I use aseptic technique when performing clinical procedures.	BIOM5 <- BIOM		0.702	2.502
I follow protocols for safe handling and disposal of contaminated linens.	BIOM6 <- BIOM		0.782	3.246
I maintain vaccination status as recommended for healthcare workers.	BIOM7 <- BIOM		0.885	2.537
I use personal protective equipment correctly and consistently.	BIOM8 <- BIOM		0.788	3.095
I follow postexposure protocols in case of potential biosecurity breaches.	BIOM9 <- BIOM		0.775	3.475
My workplace provides adequate personal protective equipment for biosecurity.	FEX1 -> FEX	Extrinsic factors (FEX)	0.811	2.157
There is continuous supervision of biosecurity practices in my hospital.	FEX2 -> FEX		0.881	2.514
The hospital has clear policies and procedures for biosecurity measures.	FEX3 -> FEX		0.864	2.463
My workplace offers regular training sessions on biosecurity protocols.	FEX4 -> FEX		0.901	2.636
I have sufficient knowledge about biosecurity measures.	FINT1 -> FINT	Intrinsic factors (FINT)	0.891	2.754
I feel confident in my ability to implement biosecurity protocols.	FINT2 -> FINT		0.808	2.734
I believe biosecurity measures are important for patient and staff safety.	FINT3 -> FINT		0.861	2.388
I actively seek opportunities to update my biosecurity knowledge.	FINT4 -> FINT		0.837	2.724
My professional experience has improved my understanding of biosecurity practices.	FINT5 -> FINT		0.845	2.303

Note: Own elaboration.

**Table 3:** Reliability and discriminant and convergent validity.

	α	CR	AVE	R^2^	FEX	FINT	BIOM
FEX	0.859	0.857	0.685		0.751		
FINT	0.853	0.932	0.662	0.725	0.546	0.658	
BIOM	0.931	0.957	0.535	0.656	0.399	0.470	0.765

**Table 4:** Testing the research hypotheses.

	Hypothesis	Path	*p* Value	2.5%	97.5%	DE	Interpretation
H_1_	FEX -> BIOM	0.319	0.000	0.123	0.522	0.102	Accepted
H_2_	FINT -> BIOM	0.520	0.002	0.305	0.714	0.105	Accepted
H_3_	FEX -> FINT -> BIOM	0.443	0.000	0.265	0.601	0.086	Accepted

## Data Availability

The datasets used and/or analyzed during the current study are available from the corresponding author upon reasonable request.

## Public Involvement Statement

No public involvement in any aspect of this research.

## Guidelines and Standards Statement

This manuscript has been written in accordance with the guidelines for the design and development of studies using a Structural Equation Modeling (SEM) approach [40] for quantitative research.
